# Polyamines release the let-7b-mediated suppression of initiation codon recognition during the protein synthesis of EXT2

**DOI:** 10.1038/srep33549

**Published:** 2016-09-21

**Authors:** Masataka Imamura, Kyohei Higashi, Katsutoshi Yamaguchi, Kiryu Asakura, Tomomi Furihata, Yusuke Terui, Toshihiko Satake, Jiro Maegawa, Kazunori Yasumura, Ai Ibuki, Tomoko Akase, Kazuhiro Nishimura, Keiko Kashiwagi, Robert J. Linhardt, Kazuei Igarashi, Toshihiko Toida

**Affiliations:** 1 Graduate School of Pharmaceutical Sciences, Chiba University, 1-8-1 Inohana, Chuo-ku, Chiba 260-8675, Japan; 2 Faculty of Pharmacy, Chiba Institute of Science, 15-8 Shiomi-cho, Choshi, Chiba 288-0025, Japan; 3 Department of Plastic and Reconstructive Surgery, Yokohama City University Medical Center, 4-57 Urafune-cho, Minami-ku, Yokohama, Kanagawa 232-0024, Japan; 4 Department of Plastic and Reconstructive Surgery, Yokohama City University Hospital, 3-9 Fukuura Kanazawa-ku, Yokohama, Kanagawa 236-0004, Japan; 5 Department of Biological Science and Nursing, Graduate School of Medicine, Yokohama City University, 3-9 Fukuura, Kanazawa-ku, Yokohama, Kanagawa 236-0004, Japan; 6 Department of Biology, Center for Biotechnology and Interdisciplinary Studies, Rensselaer Polytechnic Institute, 110 8th Street Troy, NY 12180, USA; 7 Amine Pharma Research Institute, Innovation Plaza at Chiba University, 1-8-15 Inohana, Chuo-ku, Chiba 260-0856, Japan

## Abstract

Proteoglycans (PGs), a family of glycosaminoglycan (GAG)-protein glycoconjugates, contribute to animal physiology through interactions between their glycan chains and growth factors, chemokines and adhesion molecules. However, it remains unclear how GAG structures are changed during the aging process. Here, we found that polyamine levels are correlated with the expression level of heparan sulfate (HS) in human skin. In cultured cell lines, the EXT1 and EXT2 enzymes, initiating HS biosynthesis, were stimulated at the translational level by polyamines. Interestingly, the initiation codon recognition by 43S preinitiation complex during EXT2 translation is suppressed by let-7b, a member of the let-7 microRNA family, through binding at the N-terminal amino acid coding sequence in EXT2 mRNA. Let-7b-mediated suppression of initiation codon depends on the length of 5′-UTR of EXT2 mRNA and its suppression is inhibited in the presence of polyamines. These findings provide new insights into the HS biosynthesis related to miRNA and polyamines.

Glycosaminoglycans (GAGs), a group of structurally related polysaccharides, are primarily found as the glycan moieties of proteoglycan (PG) glycoconjugates. GAGs, which include chondroitin sulfate (CS), dermatan sulfate (DS), heparin (HP), and heparan sulfate (HS), are linear, sulfated polysaccharides comprised of 50–200 repeating disaccharides of hexosamine and uronic acid^1^. Many different forms of PGs, localized on the cell surface and in the extracellular matrix, play important roles in biological processes, including cell proliferation, migration, differentiation, cell-cell crosstalk and adhesion and wound repair^2^,^3^,^4^. These biological properties frequently result from the interactions of GAGs with their protein binding-partners, and phenotypes associated with genetic disorders can be caused by mutations in genes encoding GAG biosynthetic enzymes, resulting in disturbances in the crucial biological functions of these GAGs^5^,^6^. Therefore, the structural-function relationship of GAGs has been intensively investigated to understand their physiological roles^7^,^8^. Interestingly, the sulfation pattern and extent of uronic acid epimerization are generally thought to depend on the cell or tissue type in which GAGs are expressed rather than on the nature of the core protein^9^,^10^. While we have a basic understanding of GAG biosynthesis, including extension of polysaccharide chain by glycosyltransferases (EXTs) and chain modification by epimerases and sulfotransferases^5^, the substrate specificity and the regulation of these enzymes are not well understood. For example, the structures of GAGs change during the normal embryonic development, during growth, and in aging. In CS and DS the 4-sulfation/6-sulfation (4S/6S) ratios, in articular cartilage and in skin, change during development^11^,^12^,^13^,^14^. An increase in 4S/6S ratio of CSPGs in mouse visual cortex is critical for the maturation of parvalbumin-expressing interneurons^15^. In HS, significant decreases of the levels of HS with age have been reported in human skin and Bruch’s membrane^16^,^17^. However, it remains unclear how GAG structures change during embryogenesis or in aging processes.

It has been reported that GAG biosynthesis in rabbit costal chondrocytes increased markedly after treatment of parathyroid hormone (PTH) through the up-regulation of ornithine decarboxylase (ODC), an initial and rate-limiting enzyme in the polyamine biosynthetic pathway^18^. Furthermore, GAG biosynthesis, during the differentiation of rabbit costal chondrocytes caused by PTH, depends on an increase in intracellular polyamines^19^,^20^. Polyamines (putrescine, spermidine and spermine) are present at millimolar concentrations in eukaryotic cells and are essential for the normal cell growth and differentiation^21^. Stimulation of cell growth by polyamines is mainly due to the enhancement of the protein synthesis and the expression of specific genes related to the cell growth^22^. Intracellular polyamines are strictly regulated by biosynthesis, degradation and transport^23^, however, tissue polyamine levels decrease during the aging process in animals^24^,^25^.

Here, we found that polyamine levels were well correlated with HS levels in human skin and in cell lines. The expression of HS biosynthetic enzymes, EXT1 and EXT2, was enhanced by polyamines at the level of translation. Furthermore, initiation codon recognition by 43S preinitiation complex during EXT2 translation is suppressed by let-7b, a member of the let-7 microRNA family, through the binding at the N-terminal amino acid coding sequence in EXT2 mRNA. We discovered that polyamines directly inhibit the formation of the complex between EXT2 mRNA and miRNA-induced silencing complex (miRISC) bound let-7b. These findings demonstrate that polyamines play an important role for the maturation of HS chains in PGs by enhancement of the synthesis of EXT1 and EXT2 proteins.

## Results

### Correlation between polyamines and GAGs in human skin and the effect of polyamines on wound healing in mouse skin

**Figure 1:**
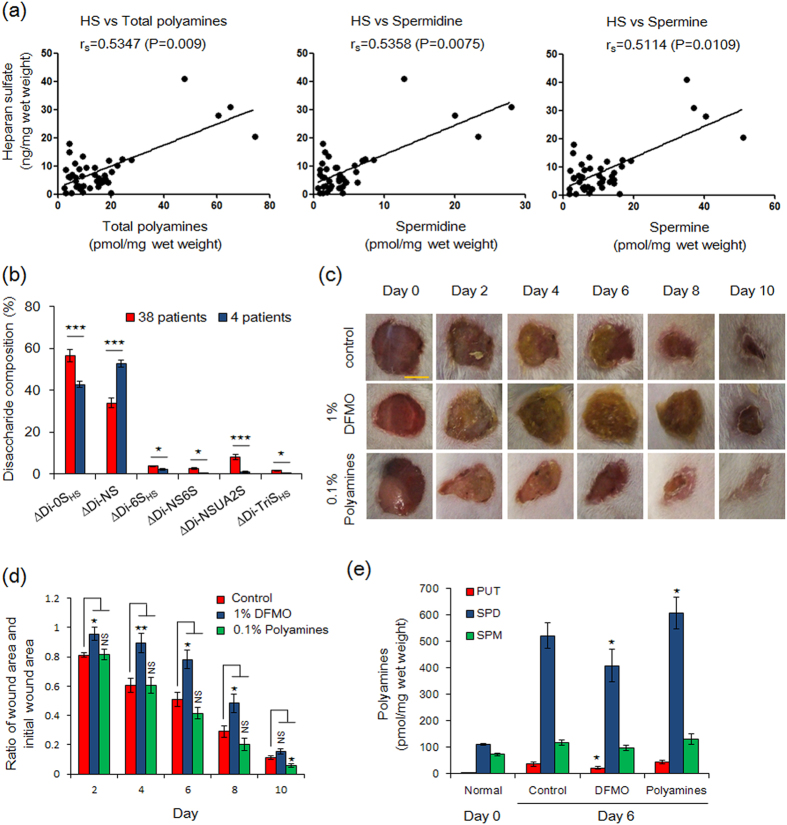
Relationship between heparan sulfate and polyamines in skin. **(a)** Correlation between HS and polyamine contents in human dermis. Median values of HS, total polyamines, spermidine and spermine were 6.2 ng/mg wet weight, 11.3, 3.4 and 7.8 pmol/mg wet weight, respectively. In four subjects increased levels of HS levels were well correlated with total polyamines, spermidine and spermine. Data of 42 subjects with breast reconstruction was evaluated by Spearman’s rank correlation analysis (r_s_ and *p* value) using GraphPad Prism^®^ Software (GraphPad Software). **(b)** Disaccharide compositions of unsaturated disaccharides of HS in dermis from four patients having a good correlation between HS and total polyamines and from other 38 patients. Data were evaluated by two-tailed unpaired Student’s *t*-tests. ^⋆⋆⋆^P < 0.005, 0.01 < ^⋆^P < 0.05. **(c,d)** Effect of polyamines on wound healing of mouse skin. Three full thickness wounds were made in individual mouse using an 8-mm biopsy punch. After wounding, hydrophilic petrolatum only (control), hydrophilic petrolatum containing 1% DFMO or 0.1% polyamines (0.075% spermidine, 0.025% spermine) were applied to wounds every day. The scale bar shown is 5 mm. Data are expressed as the mean ± s.e.m. (n = 6). ^⋆⋆^P < 0.01, 0.01 < ^⋆^P < 0.05, NS, not significant were determined by two-tailed unpaired Student’s *t*-tests. **(e)** Determination of polyamine contents in wound regions (surround skin) by HPLC. PUT, putrescine; SPD, spermidine; SPM, spermine. Data are expressed as the mean ± s.e.m. (n = 6). 0.01 < ^⋆^P < 0.05 compared to control skin.

PGs having covalently attached GAGs exhibit an important role in normal skin, maintaining mechanical strength of connective tissue and wound healing^14^,^26^,^27^,^28^. Although age-related structural alternations of GAGs have been reported^14^,^29^, the underlying mechanism for these changes remains unclear. A significant decrease of polyamine levels has also been observed in normal abdominal skin of aged mice^25^. Thus, comprehensive analysis has been performed using residual abdominal skin from patients (n = 42) undergoing breast reconstruction to discover the relationship between GAG structures and polyamines (Supplementary Tables S1–7). The disaccharide compositions of DS and HS were similar to the previously reported^14^,^17^ and no differences were observed among each individual stage (Supplementary Tables S1, S4–7). When we examined whether polyamine levels were correlated with GAGs in human skin, we observed that an increase of HS levels in the dermis correlated with total polyamine, spermidine and spermine content (Fig. 1a; Supplementary Tables S3 and S5). Since dermis samples from four patients (patients no. 7-10) showed high levels of polyamines and HS, we were able to examine their HS sulfation patterns, however, results were almost identical to other patients (Fig. 1b; Supplementary Tables S3 and S5). In the case of the epidermis, while it was impossible to analyze the samples of 19 subjects having low levels of HS, a similar relationship between HS and polyamine levels was also observed when data from the 23 remaining subjects was evaluated (Supplementary Fig. S1d; Supplementary Tables S2 and S4). These results suggest that polyamine levels are correlated with the expression level of HS in human skin. In contrast, no correlation was observed between HA and DS levels and polyamines in the epidermis or in the dermis (Supplementary Fig. S1a–c; Supplementary Tables S2, S3, S6 and S7). We also evaluated the relationships between GAGs, polyamines and age in skin. Consistent with our previous report^25^, polyamine levels in epidermis, but not in dermis, significantly decreased with age (Supplementary Fig. S2; Supplementary Tables S1–3). Unexpectedly, the levels of GAG, including DS, HA and HS and 4S/6S ratio of skin DS, did not correlate with age (Supplementary Fig. S3; Supplementary Tables S1, S4–7). We believe that this is because the ages of the 42 patients examined were restricted (38–65 years) (Supplementary Table S1). Other researchers collected cartilage samples from newborns to 85-year old patients and reported a change of 4S/6S ratio of CS up to 20 years age^11^,^13^. Since an inverse trend between age and 4S/6S ratio of DS in the epidermis is observed (Supplementary Fig. S3a; Supplementary Table S6), an increased number of samples might be required to support the observation that GAG levels correlate with age.

As mentioned above, HSPGs, including syndecan and perlecan, play a major role in tissue maintenance and wound repair^26^,^27^,^28^. It has been reported that polyamines and ornithine decarboxylase (ODC), the rate-limiting enzyme in the polyamine biosynthesis pathway, are also induced within 12 h after wounding^30^. To confirm this observation, the effect of polyamines on wound healing in mice was examined using α-difluoromethylornithine (DFMO), an irreversible inhibitor of ODC. Based on visual observations, wound closure is accomplished at around 10 days after wounding, and a delay in wound closure was observed on 1% DFMO treatment during post-wounding days 4 to 8 (Fig. 1c,d). The polyamine levels in wounded regions (surrounding skin) at days 0 and 6 were determined by HPLC, and it was found that putrescine and spermidine levels at days 6 were much higher than normal skin (day 0), suggesting the induction of ODC activity in wounded regions^30^, while significant decreases of putrescine and spermidine were observed by DFMO treatment (Fig. 1e). In addition, a slight therapeutic effect was observed on treatment with 0.1% polyamines, and spermidine content was increased compared with control tissues. These results encouraged us to investigate the physiological function of polyamines on the HS biosynthesis at the molecular levels.

### Polyamines stimulate the EXT1 and EXT2 synthesis at the level of translation

**Figure 2:**
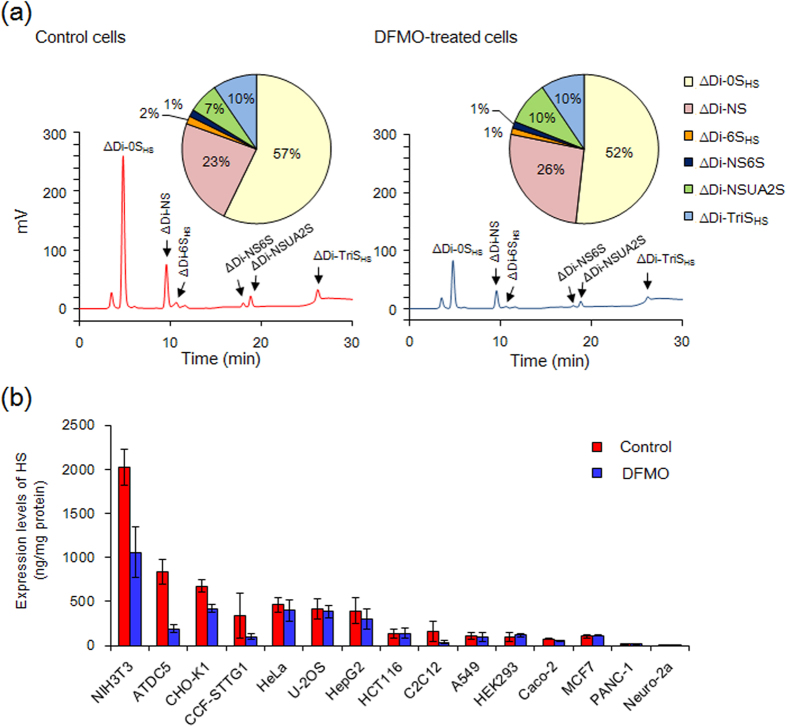
Levels and disaccharide compositions of heparan sulfate in normal and DFMO treated cells. **(a)** Chromatogram and disaccharide compositions of HS in NIH3T3 cells cultured with or without DFMO. HS purified from 1 × 10^7^ cells was treated with 1 mIU of heparinase I, II and III, and then submitted to HPLC. Note that the level of HS but not its disaccharide composition clearly changed in DFMO-treated cells. See Supplementary Methods for structures of the disaccharides. **(b)** Levels of HS in 15 cell types of untreated (control) and DFMO-treated cells. Data are expressed as the mean ± s.e.m. (n = 3). Detailed expression level and disaccharide composition of HS in 15 types of control and DFMO-treated cells are shown in Supplementary Table S9.

Effect of DFMO-induced polyamine depletion on HS expression was examined in 15 cell types where diverse sulfation patterns and levels of HS had been reported^10^. Under the cell culturing conditions used, the levels of both putrescine and spermidine were negligible (Supplementary Table S8) and on exposure to DMFO the cell numbers decreased by approximately 30% within three days as compared to control cells. The HS disaccharide compositions of the 15 cell-lines showed similar HS sulfation patterns in the DFMO-treated cells and the control cells (Supplementary Table S9). In contrast to the unaltered disaccharide compositions, the cell line dependent levels of HS decreased in DFMO-treated cells and in several cell types, particularly those having high levels of HS (Fig. 2b; Supplementary Table S9). For example, NIH3T3 dermal fibroblasts, having the highest HS level among the 15 cell types, showed a substantial decrease of HS levels on DFMO-treatment (Fig. 2a,b). Since the level of HS, but not the HS sulfation pattern, changed in DFMO-treated cells, we focused on the EXT enzymes that form the HS polysaccharide backbone to identify whether the expression levels of the genes encoding these glycosyltransferases are regulated by polyamines.

**Figure 3:**
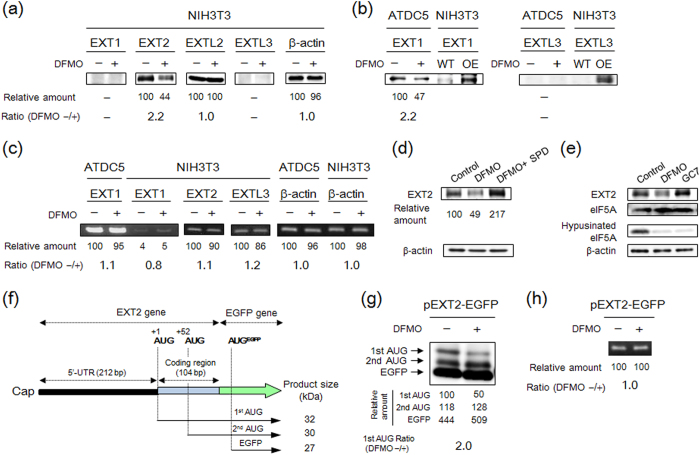
EXT1 and EXT2 synthesis are enhanced by polyamines at the level of translation. **(a)** Effect of polyamine depletion on the expression level of EXT proteins in NIH3T3 cells. For Western blotting of EXT proteins and β-actin, 30 μg (EXT1), 20 μg (EXT2), 20 μg (EXTL2), 60 μg (EXTL3) or 5 μg (β-actin) of protein of whole cell lysate, prepared from cells cultured with or without DFMO, was used. **(b)** Effect of polyamine depletion on the expression of EXT1 and EXTL3 in ATDC5 cells. For Western blotting, 30 μg (EXT1) or 60 μg (EXTL3) of protein was used. OE: over expressed. **(c)** Effect of polyamine depletion on the expression level of mRNAs of EXT gene family in NIH3T3 and ATDC5 cells. **(d)** Effects of 5 mM DFMO and/or 25 μM spermidine (SPD) on the expression level of EXT2 in NIH3T3 cells. To avoid the degradation of SPD, 1 mM aminoguanidine, an inhibitor of serum amine oxidase, was added together with SPD in culture medium for 3 days. **(e)** Effect of GC_7_, an efficient inhibitor of deoxyhypusin synthase, on the expression of EXT2 protein in NIH3T3 cells. GC_7_ (20 μM) was added to culture medium for 3 days to inhibit the hypusination for eIF5A. Note that the EXT2 expression was maintained despite an inhibition of hypusination of eIF5A by the GC_7_ treatment. For detection of the eIF5A protein, 10 μg of protein was used. **(f)** Structure of EXT2-EGFP fusion genes. Two kinds of fusion proteins (32 kDa and 30 kDa) and EGFP protein (27 kDa) can be produced by individual AUG triplets in EXT2-EGFP fusion gene. **(g)** Effect of polyamine depletion on the expression level of EXT2-EGFP fusion proteins in NIH3T3 cells. For detection of the EXT2-EGFP fusion protein, 30 μg of protein was used. (**h)** Effect of polyamine depletion on the expression level of EXT2-EGFP mRNA. Experiments were repeated in triplicate with reproducible results.

HS biosynthesis is initiated once GlcNAc is transferred by EXTL3 to the common linkage tetrasaccharides, glucuronic acid (GlcA)β1-3galactose (Gal)β1-3Galβ1-4xylose (Xyl)β1-*O*-Ser in PGs^2^,^4^. Chain elongation is then catalyzed by the EXT1/EXT2 heterodimer through the alternate addition of *N*-acetylglucosamine (GlcNAc) and GlcA residues. If GlcNAc is transferred to the linkage region by EXTL2, initiation of chain elongation is inhibited^31^. A comprehensive survey of the expression level of EXT proteins was conducted by Western blotting. Under the culturing conditions used, EXT2 protein was easily detected and a significant decrease of EXT2 expression was observed in almost all of the cells cultured with DFMO (Supplementary Fig. S4c,d). While the expression level of EXT2 protein decreased in DFMO-treated NIH3T3 cells (Fig. 3a), EXT2 mRNA levels were similar in the presence or absence of DFMO (Fig. 3c). Comparable levels of EXTL2 protein was detected in 7 cell types with or without of DFMO treatment (Supplementary Fig. S4e,f). Significant expression of EXT1 protein was only observed in murine chondrogenic ATDC5 cells (Supplementary Fig. S4a,b), and the level of mRNA in ATDC5 cells was much higher than it was in NIH3T3 cells (Fig. 3c). Furthermore, DFMO treatment decreased EXT1 protein in ATDC5 cells but did not change the level of EXT1 mRNA (Fig. 3b,c). The anti-EXTL3 antibody worked well in detecting EXTL3-overexpressing NIH3T3 cells (Fig. 3b), but EXTL3 protein was undetected in most of the cell lines tested (Supplementary Fig. S4g). Together, these results suggest that biosynthesis of EXT1 and EXT2 was enhanced by polyamines at the level of translation.

### Polyamine stimulation of EXT2 synthesis is required for the length of EXT2 mRNA 5′-untranslated region

Since polyamine stimulation of EXT2 synthesis was observed in most of the cell types (Supplementary Fig. S4c,d), additional studies were conducted at the molecular level. When the polyamine spermidine was added to rescue DFMO-treated cells, the expression level of EXT2 could be substantially recovered (Fig. 3d). It is well established that eIF5a, containing hypusine derived from spermidine, is involved in cell growth^32^. Thus, to confirm that polyamine-depletion caused the decrease of EXT2 expression in DFMO-treated cells, the effect of GC_7_, an inhibitor of active eIF5a formation, was investigated. Although the level of hypusinated eIF5a was clearly decreased, the level of EXT2 in GC_7_ treated cells was similar to control cells (Fig. 3e). These results indicate that polyamines directly stimulate EXT2 synthesis at the level of translation.

A plasmid containing 5′-untranslated region (5′-UTR) and N-terminal amino acid coding sequence (CDS) of EXT2 gene fused to the EGFP (enhanced green fluorescent protein) was next constructed to elucidate the molecular mechanism of polyamine stimulation of EXT2 synthesis (Fig. 3f). It should be noted that there are three kinds of initiation codons corresponding to first and second AUG in EXT2 gene and EGFP gene in the open reading frame. EXT2-EGFP fusion gene was transiently transformed into NIH3T3 cells, and then effect of polyamines on the synthesis of EXT2-EGFP protein was examined using anti-EGFP antibody. Interestingly, 32 kDa (first AUG), 30 kDa (second AUG) and 27 kDa (EGFP) products were produced from EXT2-EGFP fusion gene, respectively, and the expression level of first AUG only decreased in DFMO-treated cells (Fig. 3g). In contrast, expression level of EXT2-EGFP mRNA was nearly equal in cells cultured with or without DFMO (Fig. 3h). These results indicate that polyamine stimulation of EXT2 synthesis (product of first AUG) is caused at the translation initiation step.

**Figure 4:**
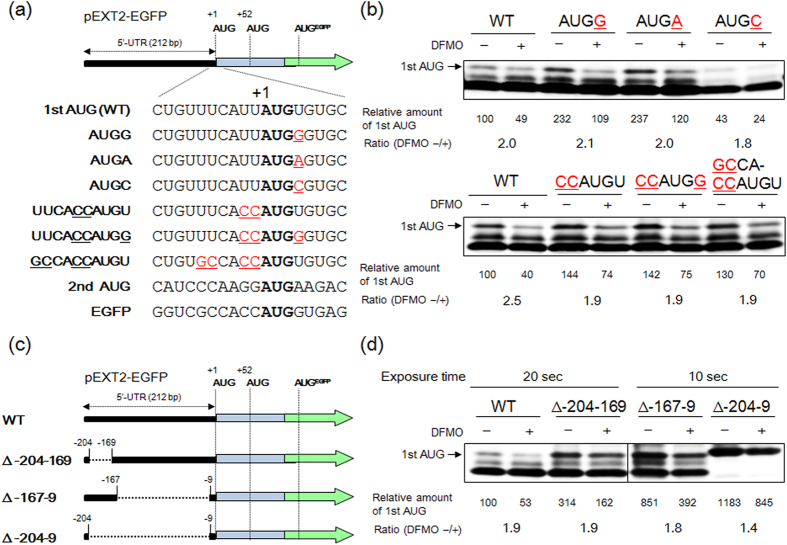
Effect of AUG codon context and 5′-UTR on the polyamine stimulation of EXT2 synthesis. **(a)** Structures of mutated AUG codon context in EXT2-EGFP fusion genes. The modified nucleotides in first AUG context are shown in red and underlined. **(b)** Effect of mutated first AUG codon context in EXT2 gene on the expression level of the first AUG product of the EXT2-fusion protein in NIH3T3 cells. **(c)** Structures of 5′-UTR deletion mutants of EXT2-EGFP fusion genes. **(d)** Effect of 5′-UTR on the expression level of first AUG product of EXT2-fusion protein in NIH3T3 cells. In the case of the 5′-UTR deletion mutants, exposure time of ECL detection was shortened compared to the WT because of a significant increase in the expression of EGFP-protein. Transfection and DFMO treatment were carried out as described under “Methods” and 30 μg of protein was used to detect the EGFP fusion protein. Experiments were repeated in triplicate with reproducible results.

In higher eukaryotes, protein synthesis is modulated at the level of initiation by the context surrounding the AUG codon (Kozak sequence) with an optimum context-GCC(A/G)CCAUGG, with a purine at the −3 and a G at the +4 positions (relative to the A of AUG codon, which is designated +1)^33^,^34^. While the first and second AUG triplets in EXT2 gene are not in poor contexts (Fig. 4a), the 43S complexes preferentially recognize the canonical AUG triplet in EGFP gene during ribosome scanning of EXT2-EGFP mRNA 5′-UTR (Fig. 3g). The first AUG codon context in the EXT2 gene was converted to the optimal context to solve the issue of leaky scanning (Fig. 4a). Although G at the +4 position of first AUG (AUGG) was important for the synthesis of first AUG product, polyamine stimulation of EXT2-EGFP synthesis was maintained in all mutants (Fig. 4b). Next, the effect of 5′-UTR length on the polyamine stimulation of EXT2 synthesis was examined. The 5′-UTR of EXT2 mRNA consists of 212 nucleotides and has a predicted secondary structure with three hairpins (Supplementary Fig. S5). Deletion mutants, corresponding to the hairpin I (positions −204 to −169), hairpin II plus III (positions −167 to −9) and hairpin I to III (positions −204 to −9), were constructed (Fig. 4c) and the effect of polyamines on EXT2-EGFP synthesis was evaluated. Surprisingly, the first AUG product of EXT2-EGFP fusion protein was preferentially synthesized as 5′-UTR length was shorter and the degree of polyamine stimulation was reduced from 1.9- to 1.4-fold (Fig. 4d) despite being unchanged at the mRNA level (Supplementary Fig. S6).

We have previously reported that polyamine stimulation of Cct2 synthesis was due to the stimulation of ribosome shunting of the stem-loop structures between take-off and landing sites, which are complementary in nucleotide sequence to 18S rRNA, during the ribosome scanning of the Cct2 mRNA 5′-UTR^35^. Moreover, a complementary sequence to 18S rRNA (CR sequence), which is distant from the initiation codon AUG in eEF1A mRNA, was required for polyamine stimulation of eEF1A synthesis^36^. Thus, effect of ribosome shunting or CR sequence on the polyamine stimulation of EXT2 synthesis was examined using site-directed mutagenesis, however, polyamine stimulations were unchanged (Supplementary Fig. S7).

Taken together, these results suggest that polyamine stimulation of preferential translation of first AUG product in EXT2 mRNA is required for the length but not nucleotide sequences of 5′-UTR for ribosome scanning.

### Initiation codon recognition by 43S preinitiation complex during EXT2 translation was suppressed by let-7b at the N-terminal amino acid coding sequence

**Figure 5:**
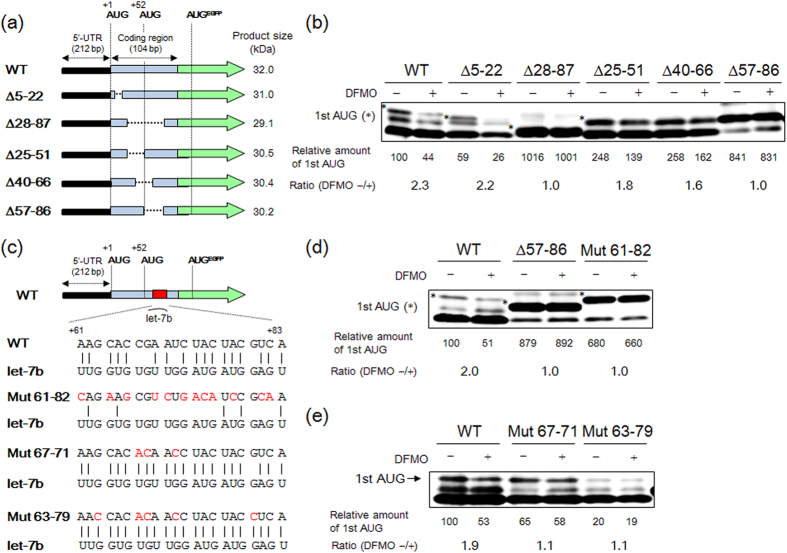
Effect of let-7b binding site in N-terminal amino acid coding sequence on polyamine stimulation of EXT2 synthesis. **(a)** Structures of deletion mutants of N-terminal CDS in EXT2-EGFP fusion genes. Based on the possible secondary structure of EXT2 mRNA obtained by Mfold (http://unafold.rna.albany.edu/), specified region of CDS was deleted (See Supplementary Fig. S8). Note that sizes of first AUG products in deletion mutants were smaller than that of WT. **(b)** Effect of N-terminal CDS on the expression levels of first AUG products (*) of EXT2-EGFP fusion proteins in NIH3T3 cells. **(c)** Sequence of let-7b binding site in EXT2-EGFP fusion gene. **(d,e)** Effects of mutations of let-7b binding site on the expression level of the first AUG products of the EXT2-EGFP fusion proteins in NIH3T3 cells. Molecular weight of the first AUG product from ∆57-86 mutant is 30.2 kDa. For detection of the EGFP fusion protein, 30 μg of protein was used. Experiments were repeated in triplicate with reproducible results.

EXT2-EGFP fusion gene was constructed by the insertion of EXT2 gene containing 5′-UTR and N-terminal CDS to the pEGFP-N1. Since N-terminal CDS is composed of 104 nucleotides, it is possible to predict a secondary structure having two stem and loops (Supplementary Fig. S8). EXT2-EGFP deletion mutants were also constructed to examine whether or not N-terminal CDS of EXT2 mRNA was involved in polyamine stimulation (Fig. 5). It should be noted that molecular weights of first AUG product from the deletion mutants were slightly reduced due to a partial deletion at the N-terminal CDS of EXT2 gene (Fig. 5a). As a result, the level of the first AUG product, EXT2-EGFP (∆ +28 to +87), but not (∆ +5 to +22), was increased compared to wild type, and the polyamine stimulation completely disappeared (Fig. 5b). Thus, we looked for the polyamine-sensitive site and found that 29 base pairs corresponding to the +57 to +86 positions in N-terminal CDS was necessary for the polyamine stimulation of EXT2 synthesis (Fig. 5b). The similarity with miRNA sequences, identified in the +57 to +86 positions, was searched using miRBase (http://www.mirbase.org), and only let-7b, a member of the let-7 microRNA family^37^, was identified.

MicroRNA (miRNA) is a small non-coding RNA, containing approximately 22 nucleotides in length, which can catalyze the repression of translation initiation and mRNA degradation through the binding to the 3′-UTR of target mRNAs^38^,^39^. Translational repression of target mRNA by miRNA is required for the association with an Argonaute protein (AGO) and GW182-family protein in effector complexes known as miRNA-induced silencing complexes (miRISCs), however, the detailed translation repression mechanism, particularly the inhibition of translation initiation, has been under investigation^39^. In addition, there are several reports that miRNAs can also target mRNAs within their CDS (especially, the middle of CDS) and decrease their translation^40^,^41^,^42^,^43^,^44^,^45^. The potential let-7b target site was mutated to disrupt base-pairing interaction with let-7b to validate the interaction between let-7b and N-terminal CDS (Fig. 5c). Surprisingly, the first AUG product was preferentially synthesized when EXT2-EGFP Mut (+61 to +82) was transfected, and the polyamine stimulation completely disappeared (Fig. 5d). These results strongly suggest that initiation codon recognition by 43S preinitiation complex during EXT2 translation is suppressed by let-7b through the binding at the N-terminal CDS in EXT2 mRNA. Since imperfect base pairing of 3′-end of let-7b and EXT2 mRNA was suggested (Fig. 5c), the effect of perfect base pairing of let-7b/mRNA on polyamine stimulation of EXT2 synthesis was next examined. As previously reported^46^, perfect base pairing of let-7b/mRNA in EXT2-EGFP Mut (+67 to +71) and Mut (+63 to +79) exhibited the decrease of the expression level of the first AUG product, and the polyamine stimulation completely disappeared (Fig. 5e). These results demonstrate that polyamine stimulation of EXT2 translation initiation at the first AUG codon is related to the imperfect base pairing of 3′-end of let-7b/EXT2 mRNA hybrids.

### Polyamines inhibit the binding let-7b/miRISC complex to EXT2 mRNA

**Figure 6:**
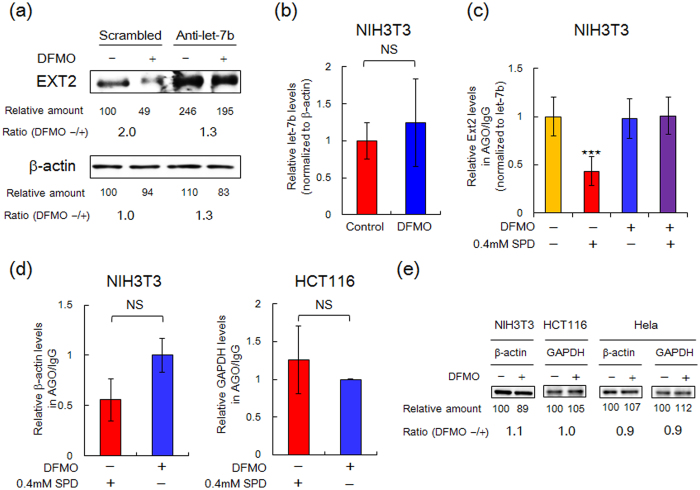
Direct inhibition of let-7b binding by spermidine to its target sequence. **(a)** Effect of anti-let-7b on the expression level of EXT2 protein in NIH3T3 cells. The microRNA inhibitor for let-7b or negative control A (scrambled) were transfected to NIH3T3 cells for 72 h in the presence or absence of 5 mM DFMO. **(b)** Effect of polyamine depletion on the expression level of let-7b in NIH3T3 cells was examined by qPCR. The level of let-7b expression in control cells was defined as 1.0. NS, not significant, two-tailed unpaired Student’s *t*-tests. **(c,d)** Immunoprecipitation (IP) of miRNA/EXT2 mRNA **(c)**, β-actin or GAPDH mRNAs **(d)** complexes associated with Argonate proteins (Ago1, Ago2 or Ago3) from NIH3T3 or HCT116 cell extract. Precipitated miRNA/mRNA complexes were treated with proteinase K, and resulting free mRNAs were converted to cDNA and quantified by qPCR. The results obtained with Ago-IP were normalized by mouse IgG-IP as a negative control. **(c)** In this pull-down assay system, since precipitated let-7b by Ago-IP was also detectable, EXT2 mRNA was further normalized to let-7b. Data are expressed as the mean ± s.e.m. of three independent experiments and data was by one-way ANOVA with Tukey-Kramer post-test using GraphPad Prism^®^ Software (GraphPad Software). ^⋆⋆⋆^P < 0.005. **(d)** In case of values of β-actin and GAPDH, both of which are obtained from three independent experiments are expressed as the mean ± s.e.m. Data were evaluated by two-tailed unpaired Student’s *t*-tests, however no significant difference was observed. **(e)** Effect of polyamine depletion on the expression of β-actin and GAPDH.

We have previously reported that selective structural changes by spermidine in the bulged-out region of OppA mRNA and in the acceptor stem of rat liver tRNA^Ile^ are well correlated with polyamine stimulation of OppA synthesis and Ile-tRNA formation^47^. Therefore, we speculated that imperfect base pairing of 3′-end of let-7b and EXT2 mRNA, containing bulged-out region, was important for the polyamine stimulation of EXT2 synthesis. The effect of anti-miRNA antisense oligonucleotides on the polyamine stimulation of EXT2 synthesis was examined to elucidate the detailed mechanism. The expression level of EXT2 protein was significantly increased by anti-let-7b treatment, and the polyamine stimulation was greatly reduced, suggesting the physiological impact of let-7b regulation at the N-terminal CDS for the expression of intact EXT2 (Fig. 6a).

Polyamine depletion by DFMO treatment induced the expression of miRNA let-7i through the down-regulation of Lin28, an inhibitory protein of the let-7 biogenesis through pre-miRNA processing^48^,^49^. However, expression level of let-7b was nearly equal in NIH3T3 cells cultured with or without DFMO (Fig. 6b). Typically, the miRNA level is normalized using U6 snRNA, however, because U6 snRNA but not EXT2 and β-actin mRNA in DFMO-treated cells decreased to approximately 30% of control, β-actin mRNA was used in place of U6 RNA for normalization and quantification of let-7b expression.

Next, the physical interaction of let-7b with endogenous EXT2 mRNA in cells cultured with or without DFMO was evaluated using a pull-down assay with anti-Ago1/2/3 antibody and protein G magnetic beads. After the preparation of cell lysates, binding of EXT2 mRNA to miRISC-bound let-7b precipitated by anti-Ago1/2/3 antibody and evaluated by qPCR. Unexpectedly, the EXT2 mRNA/let-7b complex precipitated in cell lysates from cells cultured with or without DFMO were nearly equal (Fig. 6c; columns 1 and 3). This result might be explained by the affinity of miRNA/mRNA complex being higher than that of spermidine/mRNA complex. In fact, the reported *Kd* value for binary hAgo2/guide RNA complex to complementary target RNA is 0.2 nM^50^, in contrast *Kd* value for spermidine to double stranded RNA with bulged-out region is 0.4 mM^47^. When exogenous spermidine was added to cell lysate from control cells, significant inhibition of the interaction between let-7b and EXT2 mRNA was observed (Fig. 6c; column 2). In contrast, exogenous spermidine was unable to dissociate EXT2 mRNA/let-7b complex formed in cell lysates from cells cultured with DFMO (Fig. 6c; column 4). These results suggest that spermidine can directly inhibit EXT2 mRNA/let-7b complex formation in cells.

Expression of GAPDH and β-actin are regulated by miR-644a at the 3′-UTR^51^. Despite a slight decrease of the binding of β-actin mRNA to miRISC by the addition of exogenous spermidine (Fig. 6d), expression of both proteins were almost same in cells cultured with or without DFMO (Fig. 6e). In light of this, it is suggested that polyamine stimulation of EXT2 synthesis is required for the inhibition of the formation of EXT2 mRNA/let-7b complex by spermidine at the N-terminal CDS but not 3′-UTR.

## Discussion

We have previously found that polyamines exist mainly as polyamine-RNA complexes in *E. coli*, bovine lymphocytes and rat liver, and the expression level of some genes are enhanced by polyamines at the level of translation. We proposed that a set of genes whose expression is enhanced by polyamines at the level of translation can be referred to as a “polyamine modulon”^21^,^22^. In general, translational efficiencies of polyamine modulon are low because there are complementary sequences to 18S rRNA required for the ribosome arrest in 5′-UTR of Cct2 and eEF1A mRNA and it has been suggested that polyamines cause structural changes in the bulged-out structures of mRNA, tRNA and ribosomal RNA, facilitating their translation^35^,^36^,^47^. In this study, EXT1 and EXT2 were determined to represent a new polyamine modulon. Interestingly, initiation codon recognition by 43S preinitiation complex during EXT2 synthesis was suppressed by let-7b through the binding at the N-terminal CDS in EXT2 mRNA (Fig. 5) and let-7b/EXT2 mRNA complex formation was directly inhibited by polyamines (Fig. 6). Furthermore, polyamine stimulation of EXT2 synthesis depends on the length of 5′-UTR of EXT2 mRNA (Fig. 4d), indicating that suppression of initiation codon recognition by miRISC occurred during ribosome scanning. These results are similar to the other reports that cap-mediated, but not IRES-mediated, translation initiation is sensitive miRNA-mediated repression^39^,^52^.

It has been reported that polyamine depletion by DFMO treatment or eIF5A1/2 knockdown in HCT116 cells induced the down-regulation of Lin28, a post-transcriptional regulatory protein of the let-7 family of miRNAs^37^, and consequently let-7i expression increased^48^. However, the expression level of let-7b was nearly equal in NIH3T3 cells cultured in the presence or absence of DFMO (Fig. 6b). Heo *et al*.^49^ reported that tetra-nucleotide sequence motif (GGAG) in the terminal loop of let-7 precursor was important for the recognition of Lin28, and if GGAG motif was mutated to GAGG or AAAG, the interaction between Lin28 and pre-let-7 was weakened. Although a binding assay was not utilized, in studies of the binding between Lin28 and pre-let-7 having GAAG motif (let-7b type) in the terminal loop, it was proposed that the recognition of let-7b was weaker than with other let-7 families^49^. Furthermore, hypusinated eIF5a was not involved in EXT2 synthesis (Fig. 3e). For these reasons, we concluded that maturation of let-7b was not involved in polyamine stimulation of EXT2 synthesis.

During ribosome scanning, it was established that eIF1 functions in the critical role of initiating codon recognition^34^, however, there are no reports of the contribution of eIF1 for the repression of translation by miRISCs. Meijer *et al*.^52^ reported that miRNA-mediated translational repression is achieved by the recruitment of the DEAD-box helicase protein eIF4AII by miRSCs. The RNA binding protein HuD, an inhibitor of miRISC-induced dissociation eIF4As protein, was recently reported^53^. However, it is not possible to explain the polyamine stimulation of translation through the up-regulation of HuD activity because polyamines mainly exist within cells as polyamine-RNA complexes^21^. Furthermore, HuD and eIF4As are ubiquitously utilized in all translation for many kinds of gene expression^34^,^53^. Therefore, we have focused the effect of polyamines on the imperfect base pairing of 3′-end of let-7b/EXT2 mRNA hybrids (Fig. 6c), however, it remains unclear why formation of let-7b/EXT2 mRNA complex but not miR644/β-actin or GAPDH mRNA complex was significantly inhibited by polyamines (Fig. 6d,e). Interestingly, when the sequences near the let-7b binding site (positions +25 to +51 and +40 to +66) were removed, the degree of let-7b suppression decreased (increased at the level of the first AUG product). This was despite an unchanged level of polyamine stimulation (Fig. 5b), suggesting that the formation of miRNA/mRNA complexes might be influenced by tertiary structure of the target mRNA. Indeed, the let-7b binding site is located at the internal loop structure of N-terminal CDS in EXT2 mRNA (Supplementary Fig. S8). Thus, it is likely that specific inhibition, of miRNA function by polyamines, depends on the tertiary structure of RNA containing miRNA binding site or its position in the target mRNA. Experiments are in progress to clarify the polyamine inhibition mechanism for the formation of miRNA/mRNA complexes.

There are several reports of HSPGs, including syndecan and perlecan, playing a major role in tissue maintenance and wound repair^26^,^27^,^28^, and polyamines and ODC are also induced within 12 h after wounding^30^. Based on the results of previous studies and Fig. 1c, we speculate that maintenance of high levels of polyamine contribute, at least in part, to the mechanical strength of connective tissue of normal skin and wound healing through the maturation of HS chains in PGs. Although EXT1 and EXT2 were determined to be polyamine modulons, decreased levels of HS in DFMO-treated cells was observed, while expression levels of HS in control cells were higher (Fig. 2b). It should be noted that HS expression on the plasma membrane is also regulated by lysosomal degradation and HS-degrading enzyme heparanase^54^,^55^. In general, plasma membrane bound HSPG, biosynthesized in the Golgi, is destined for loss from the cell surface either through the release into the medium (55%, with *t*_1/2_ of 2.5 h) or through internalization (45%, with with *t*_1/2_ of 6.2 h)^54^. In addition, HS turnover depends on culturing conditions^56^. Therefore, we hypothesize that the high degradation activity of HS, exhibited in the disappearance of polyamine stimulation, impact cells having low HS level. In addition to HS degradation, it also remains to be clarified whether or not EXTL3 gene is polyamine modulon. Unfortunately, effect of polyamines on the EXTL3 synthesis cannot be easily examined because expression level of EXTL3 is considerably lower than that of other EXT family members (Supplementary Fig. S4g). Since overexpression of EXTL3 protein was successfully conducted when cDNA containing CDS of EXTL3 gene was transfected in NIH3T3 cells, it was thought that the efficiency of transcription or translation system of EXTL3 gene was very low. Indeed, the length of 5′-UTR in EXTL3 mRNA is 664 nucleotides and GC contents are 52%. Thus, further experiments are required.

In conclusion, our results demonstrate that expression levels of HS were well correlated with polyamine contents in human skin. In addition, polyamine stimulated wound healing in mouse skin. EXT1 and EXT2 synthesis was enhanced by polyamines at the level of translation. Recognition of the first AUG triplets by 43S preinitiation complex during EXT2 translation was suppressed by let-7b at the N-terminal amino acid coding sequence and polyamines directly suppressed the formation of let-7b/EXT2 mRNA complex. These results indicate that polyamines play an important role for the maturation of HS chains in PGs.

## Methods

### Materials

Actinase E was purchased from Kaken Pharmaceutical Co., Ltd., Tokyo, Japan. Chondroitinase ABC (ChaseABC) from *Proteus vulgaris*, chondroitinase ACII (ChaseACII) from *Arthrobacter aurescens*, Heparinase I, heparinase II, heparinase III from *Flavobacterium heparinum*, unsaturated disaccharides of CS (ΔDi-0S, ΔDi-4S, ΔDi-6S, ΔDiUA-2S, ΔDi-diS_E_, ΔDi-diS_B_, ΔDi-diS_D_, ΔDi-TriS), and of HS (ΔDi-0S_HS_, ΔDi-NS, ΔDi-6S_HS_, ΔDi-NS6S ΔDi-NSUA2S, ΔDi-TriS_HS_) were purchased from Seikagaku Corp., Tokyo, Japan. *N*^1^-Guanyl-1,7-diaminoheptane (GC_7_), an inhibitor of deoxyhypusine synthase^32^, was a kindly supplied by Dr. M. H. Park, National Institutes of Health.

### Ethics statement

The natural part of abdominal walls were obtained from patients with immediate breast reconstruction with stage I (n = 5) or stage II (n = 8), and delayed breast reconstruction with stage I (n = 4) or stage II (n = 25). Informed consent was given by each participant, and our study was approved by the ethics committees of Graduate School of Medicine, Yokohama City University, and of Graduate School of Pharmaceutical Sciences, Chiba University. Experiments were conducted in accordance with the Declaration of Helsinki principles. Animal experiments were approved by the Institutional Animal Care and Use Committee of Chiba University and carried out according to the guidelines for Animal Research of Chiba University.

### Quantitative analysis of GAGs and polyamines from human skin and cultured cells

Epidermis and dermis samples from abdominal skins were prepared by Dispase II (Sigma) treatment according to the method of Huang *et al*.^57^. Epidermis or dermis samples were cut into small pieces and used for the determination of GAGs and polyamine analysis (Supplementary Table S1). Dried skin samples were homogenized with 4-volumes of acetone overnight, and then the precipitates obtained by centrifugation were proteolyzed at 45 °C with actinase E (10 mg/g dry powder) in 50 mM Tris acetate (pH 8.0) for 18 h. In the case of cultured cells, 1 ×  10^7^ cells were freeze-dried overnight and treated with actinase E (0.25 mg/mL) in 400 μL of 50 mM Tris-acetate buffer (pH8.0) at 45 °C for 3 days. Microscale isolation of GAGs was performed according to the method of Zhang *et al*.^58^. Briefly, the filtered extracts were purified by a Vivapure Q mini H spin column (Sartorius AG) centrifugation, and then columns were washed three-times with 450 μL of 0.2 M NaCl. Crude GAGs were then eluted from the column by washing two-times with 500 μL of 16% NaCl. The GAGs were precipitated from the supernatant by addition of four-volumes of cold methanol for 16 h at 4 °C and were recovered by centrifugation at 11,000 × *g* for 30 min. The whole GAG samples were incubated in the reaction mixture (35 μL) containing 28.6 mM Tris-acetate (pH 8.0) and 50 mIU of Chase ABC (and Chase ACII). After 16 h at 37 °C, depolymerized samples were boiled and evaporated, unsaturated disaccharides of CS/DS were collected by Amicon Ultra centrifugal Filter 30 K device (EMD Millipore). The remaining HS samples in filters of spin column were transferred to new microtubes and incubated in 16 μL of reaction mixture (pH 7.0) containing 1 mIU heparinase I, 1 mIU heparinase II, 1 mIU heparinase III, 31.3 mM sodium acetate and 3.13 mM calcium acetate for 16 h at 37 °C. Unsaturated disaccharides analysis by a reversed phase ion-pair chromatography with sensitive and specific post-column detection and HPLC analysis for polyamines were performed as described under Supplementary Methods. Protein contents were determined by the method of Lowry *et al*.^59^.

### Wound model mouse

Eight-week-old of six male ddY mice (Japan SLC, Inc), weighing 35 g, were used in this study. The animals were fed on a laboratory chow *ad libitum*. The backs of mice were shaved and three full thickness wounds were made using a disposable biopsy punch (diameter 8 mm; Kai Industries) under isoflurane anesthesia. After wounding, hydrophilic petrolatum (Maruishi Pharmaceutical Co., Ltd) only (control) and hydrophilic petrolatum containing 1% DFMO or 0.1% polyamines (0.075% spermidine and 0.025% spermine) were applied to wounds every day. The sizes of wounds at the specified day were calculated by ImageJ imaging software. Determination of polyamine contents in wound regions were performed as described under Supplementary Methods.

### Cell culture

CHO-K1, CCF-STTG1, HepG2, HEK293, Caco-2, MCF7 and PANC-1 were purchased from ATCC. ATDC5 were purchased from RIKEN Cell Bank, in Japan. U-2OS, HCT116, A549 and Neuro-2a were kindly supplied by Dr. Murai (The Jikei University School of Medicine, Japan), Dr. Fukumoto (Chiba University, Japan), Dr. Nakamura (Chiba University, Japan) and Dr. Kitagawa (Kobe Pharmaceutical University, Japan), respectively. Cells were cultured in Dulbecco’s modified Eagle’s medium supplemented with 10% fetal bovine serum (FBS), 100 units/mL penicillin G and 50 units/ml streptomycin in an atmosphere of 5% CO_2_/95% air at 37 °C. A three-fold greater number of cells were cultured in DMEM medium in the presence of DFMO for 3 days to make polyamine-depleted cells. The concentration of DFMO used in this study was listed in Supplementary Table S8.

### Western Blot Analysis

Western blot analysis was performed by the method of Nielsen *et al*.^60^ using Amersham^TM^ ECL Select^TM^ Western Blotting Detection System (GE Helthcare Life Science). Antibodies used in this study are listed in Supplementary Table S10. The level of protein was quantified by an ImageQuant^TM^ LAS 4000 (GE Helthcare Life Science). Protein contents were determined by the method of Lowry *et al*.^59^.

### Plasmids

PCR was performed using the following primer sets, P1 and P2, P3 and P4, P5 and P6, respectively, and cDNA prepared from total RNA isolated as mentioned later was used as a template to amplify the EXT1 (2270 bp), EXT2 (336 bp) and EXTL3 (2790 bp) genes. All primers used for the construction of pEXT2-EGFP, pEXT2-EGFP mutants, pcDNA3.1/EXT1-*myc*-His and pcDNA3.1/EXTL3-*myc*-His are listed in Supplementary Table S11. The amplified EXT2 gene containing 5′-UTR and N-terminal CDS was digested with EcoRI and BamHI, and inserted into the same restriction site of pEGFP-N1 (Takara Bio Inc.). The amplified EXT1 and EXTL3 genes correspond to open reading frame were digested with BamHI and EcoRI (EXT1), KpnI and XhoI (EXTL3), respectively, and inserted into the same restriction site of pcDNA3.1/*myc*-His A (Thermo Fisher Scientific Inc.). Overlap extension PCR was performed using primers listed in Supplementary Table S11 and pEXT2-EGFP was used as a template to make pEXT2-EGFP mutants [pEXT2 (AUGG)-EGFP, pEXT2 (AUGA)-EGFP, pEXT2 (AUGC)-EGFP, pEXT2 (CCAUGU)-EGFP, pEXT2 (CCAUGG)-EGFP, pEXT2 (GCCACCAUGU)-EGFP, pEXT2 (∆-204-169)-EGFP, pEXT2 (∆-167-9)-EGFP, pEXT2 (∆-204-9)-EGFP, pEXT2 (NC-167-162)-EGFP, pEXT2 (NC-16-9)-EGFP, pEXT2 (NC-37-29)-EGFP, pEXT2 (∆5-22)-EGFP, pEXT2 (∆28-87)-EGFP, pEXT2 (∆25-51)-EGFP, pEXT2 (∆40-66)-EGFP, pEXT2 (∆57-86)-EGFP, pEXT2 (Mut61-82)-EGFP, pEXT2 (Mut67-71)-EGFP, pEXT2 (Mut63-79)-EGFP]. pEXT2 (SD-20)-EGFP and pEXT2 (SD-10)-EGFP were constructed using pEXT2 (NC-37-29)-EGFP as a template and primers listed in Supplementary Table S11. The plasmid sequences were confirmed by DNA sequence service (Eurofins Genomics). Possible secondary structure of EXT2 mRNA was obtained by the method of Zuker^61^.

### Transfection of plasmids and miRNA inhibitor

Transfection of plasmids into NIH3T3 cells was performed according to the method of Fukumoto *et al*.^62^ with minor modifications. Briefly, 1 μg of plasmids was mixed with 6 μg of polyethylenimine (Polysciences, Inc.) in 100 μL of buffer (20 mM sodium lactate, 150 mM NaCl (pH 4.0)) and stand for 20 min at room temperature, and then 500 μL of Opti-MEM^®^I (Thermo Fisher Scientific Inc.) was added. The 1.2 × 10^5^ cells in 6 well plates were cultured with or without 5 mM DFMO for 12 h. After changing the medium with a fresh one containing FBS, cells were transfected with 600 μL of plasmid/polyethylenimine complex in Opti-MEM and cultured in DMEM containing FBS with or without 5 mM DFMO for 8 h. After replacing the culture medium with fresh one, cells were cultured with or without 5 mM DFMO for further 16 h.

Tranfection of miRCURY LNA™ miRNA inhibitor for *let-7b* (ACCACACAACCTACTACCTC) or negative control A (TAACACGTCTATACGCCCA) (Exiqon Inc.) were performed with lipofectamine 2000 (Thermo Fisher Scientific Inc.) according to the accompanying manual.

### Measurement of mRNA and let-7b

Total RNA was isolated with RNeasy Mini Kit (QIAGEN GmbH). DNase treatment of RNA samples prior to reverse transcription was performed using RQ1 RNase-Free DNase (Promega). After removal of degraded DNA by Amicon Ultra −0.5 mL Centrifugal Filters 3 K device (EMD Millipore), synthesis of the first-strand cDNA from total RNA was performed using SuperScript™ II Reverse Transcriptase (Thermo Fisher Scientific Inc.) according to the accompanied manual. Semi-quantitative RT-PCR was performed using primers listed in Supplementary Table S11, and cDNA as a template. The expression level was analyzed by ImageJ imaging software.

Total RNA was isolated with miRNeasy Mini Kit (QIAGEN GmbH) to evaluate the expression level of let-7b. The first-strand cDNA was prepared using Mir-X™ miRNA First-Strand synthesis Kit (Takara Bio Inc.) according to the accompanied manual. Since RNA molecules are polyadenylated and transcribed in this system, quantitative PCR (qPCR) with SYBR Advantage qPCR Premix (Takara Bio Inc.) are available for the detection not only let-7b but also EXT2, β-actin and GAPDH mRNA using primers listed in Supplementary Table S11. The transcript levels were caluculated by 2^−ΔΔCt^ method. Difference of relative EXT2 levels was determined by analysis of variance (one-way ANOVA with Tukey-Kramer post-test).

### miRNA/mRNA immunoprecipitation assay with anti-Ago1/2/3 antibody

miRNA/mRNA immunoprecipitation was performed by using miRNA Target IP kit (Active Motif) according to the accompanying manual with minor modifications. Antibody conjugation to protein G beads was performed as follows. Protein G magnetic beads (25 μL) were mixed with 100 μL of BSA solution for 10 min, and then the tubes were placed on a magnet to pellet that beads. After removal of the supernatant, 50 μL of wash buffer containing 2.5 μg of antibody was incubated for 30 min.

NIH3T3 cells were cultured in the presence or absence of 5 mM DFMO for 3 days. Cells were lysed in 150 μL of complete lysis buffer and freezed at −80 °C. Supernatant (70 μL) obtained by the centrifugation at 15,000 × *g* and 450 μL of immunoprecipitation buffer were added to the protein G magnetic beads conjugated with 2.5 μg of anti-Ago1/2/3 antibody or negative control anti-IgG antibody and mixed. After incubation for overnight at 4 °C, samples were collected and treated with proteinase K to digest protein for 30 min at 55 °C. Preparations of first-strand cDNAs and qPCR for the detection of RNAs in precipitated RISCs were mentioned above. The result of Ago-IP was normalized by that of mouse IgG-IP as a negative control.

## Additional Information

**How to cite this article**: Imamura, M. *et al*. Polyamines release the let-7b-mediated suppression of initiation codon recognition during the protein synthesis of EXT2. *Sci. Rep*. **6**, 33549; doi: 10.1038/srep33549 (2016).

## Supplementary Material

Supplementary Information
