# Supplementary material for: Polyamines release the let-7b-mediated suppression of initiation codon recognition during the protein synthesis of EXT2
Source: Sci Rep. 2016 Sep 21;6:33549. doi: 10.1038/srep33549 (PMC5030709; doi:10.1038/srep33549)
Supplement: Supplementary Information [file srep33549-s1.doc]

**SUPPLEMENTARY INFORMATION**

**Polyamines release the let-7b-mediated suppression of initiation codon recognition during the protein synthesis of EXT2**

Masataka Imamura^1^, Kyohei Higashi^1^, Katsutoshi Yamaguchi^1^, Kiryu Asakura^1^, Tomomi Furihata^1^, Yusuke Terui^2^, Toshihiko Satake^3^, Jiro Maegawa^4^, Kazunori Yasumura^3^, Ai Ibuki^5^, Tomoko Akase^5^, Kazuhiro Nishimura^1^, Keiko Kashiwagi^2^, Robert J. Linhardt^6^, Kazuei Igarashi^1, 7^ & Toshihiko Toida^1^

*^1^Graduate School of Pharmaceutical Sciences, Chiba University, 1-8-1 Inohana, Chuo-ku, Chiba 260-8675, Japan.*

*^2^Faculty of Pharmacy, Chiba Institute of Science, 15-8 Shiomi-cho, Choshi, Chiba 288-0025, Japan.*

*^3^Department of Plastic and Reconstructive Surgery, Yokohama City University Medical Center, 4-57 Urafune-cho, Minami-ku, Yokohama, Kanagawa 232-0024, Japan.*

*^4^Department of Plastic and Reconstructive Surgery, Yokohama City University Hospital, 3-9 Fukuura Kanazawa-ku, Yokohama, Kanagawa* *236-0004, Japan.*

*^5^Department of Biological Science and Nursing, Graduat School of Medicine, Yokohama City University, 3-9 Fukuura, Kanazawa-ku, Yokohama, Kanagawa 236-0004, Japan.*

*^6^Department of Biology, Center for Biotechnology and Interdisciplinary Studies, Rensselaer Polytechnic Institute, 110 8th Street Troy, NY 12180, USA.*

*^7^Amine Pharma Research Institute, Innovation Plaza at Chiba University, 1-8-15 Inohana, Chuo-ku, Chiba 260-0856, Japan.*

Correspondence and requests for materials should be addressed to K. H. (email: higase@faculty.chiba-u.jp)

**Supplementary Figure S1. Relationships between GAGs and polyamines in human skin.**  Correlation between polyamines and DS **(a)**, 4S/6S ratio of DS **(b)**, HA **(c)**, or HS **(d)**. Data of 42 subjects with breast reconstruction was evaluated by Spearman’s rank correlation analysis (r_s_ and *p* value) using GraphPad Prism® Software (GraphPad Software). Median values of DS, 4S/6S ratio of DS (n=37), HA (n=41) and HS in epidermis were 2.0 ng/mg wet weight, 6.6, 1.8 ng/mg wet weight and 0.2 ng/mg wet weight, respectively. In case of epidermis HS **(d)**, data of 23 subjects were also evaluated, and median value was 2.9 ng/mg wet weight. Median values of DS, 4S/6S ratio of DS and HA in dermis were 140.5 ng/mg wet weight, 18.6, 304.8 ng/mg wet weight, respectively.

**Supplementary Figure S2. Relationships between polyammines and age in human skin.** Median values of polyamines in epidermis and dermis were 153.3 pmol/mg wet weight, 11.3 pmol/mg wet weight, respectively. Data of 42 subjects with breast reconstruction was evaluated by Spearman’s rank correlation analysis (r_s_ and *p* value) using GraphPad Prism® Software (GraphPad Software).

**Supplementary Figure S3. Relationships between GAGs and age in human epidermis (a) and dermis (b).** Data of 42 subjects with breast reconstruction were evaluated by Spearman’s rank correlation analysis (r_s_ and *p* value) using GraphPad Prism® Software (GraphPad Software). In case of epidermis HS, data of 23 subjects were also evaluated.

**Supplementary Figure S4. Effect of polyamine depletion on the expression level of EXT1 (a,b), EXT2 (c,d), EXTL2 (e,f), EXTL3 (g) and β-actin (h) in several kinds of cells.** For Western blotting of EXT proteins and β-actin, 30 μg (EXT1), 20 μg (EXT2), 20 μg (EXTL2), 60 μg (EXTL3) or 5 μg (β-actin) of protein of whole cell lysate prepared from cells cultured with or without DFMO was used.

**Supplementary Figure S5. Possible secondary structure of 5’-untranslated region in EXT2 mRNA.**

**Supplementary Figure S6. Effect of polyamine depletion on the expression level of mRNAs in EXT2-EGFP mutants.** Mutated EXT2-EGFP genes were transfected to NIH3T3 cells and then cultured in medium with or without 5 mM DFMO. Preparation of first stranded cDNA and semi-quantitative RT-PCR were performed as described under “Methods”.

**Supplementary Figure S7. Relationship between polyamine stimulation and complementary sequences to 18S rRNA.**

**(a)** The possible secondary structure of the 5’-UTR of EXT2 and complementary putative contact sequence in 18S rRNA (shown in blue) are shown. The EXT2-EGFP genes whose sequences (-167 to -162, -37 to -29 or -16 to-9) were converted to non-complementary sequence to 18S rRNA were termed as NC-167-162, NC-37-29 and NC-16-9, respectively. **(b)** The expression levels of EXT2-EGFP synthesized from NC-167-162 and NC-16-9 were examined by Western blotting. **(c)** Nucleotide sequences of mutants at the region of CR sequence of EXT2-EGFP genes are shown. The EXT2-EGFP genes in which the position of CR sequence shifted were termed as SD-20 and SD-10, respectively. CR sequence is shown in red. **(d)** The expression levels of EXT2-EGFP synthesized from NC-37-29, SD-20 and SD-10 were examined by Western blotting.

**Supplementary Figure S8. Possible secondary structure of N-terminal amino acid coding sequence in EXT2 mRNA.**

**Figure S9. Uncropped versions of immunoblots and RT-PCR referring to Fig. 3a, 3b, 3d, 3e and 3g.**

**Figure S10. Uncropped versions of immunoblots referring to Fig. 4b and 4d; Fig. 5b, 5d and 5e.**

**Figure S11. Uncropped versions of immunoblots referring to Fig. 6a and 6e.**

**Supplementary Table S1.** Grade of breast cancer, age of patients and wet weights of abdominal skins used in this study.

**Supplementary Table S2.** Polyamine contents in human epidermis.

**Supplementary Table S3.** Polyamine contents in human dermis.

**Supplementary Table S4.** Level and composition of disaccharides in HS in human epidermis.

**Supplementary Table S5.** Level and composition of disaccharides in HS in human dermis.

**Supplementary Table S6.** Level and composition of disaccharides in DS and HA in human epidermis.

**Supplementary Table S7.** Level and composition of disaccharides in DS and HA in human dermis.

**Supplementary Table S8.** Determination of polyamine contents in cells cultured with or without DFMO. Experiments are expressed as the mean of three independent experiments.

**Supplementary Table S9.** Level and composition of unsaturated disaccharides in HS of control and DFMO treated cells**.** Experiments are expressed as the mean of three independent experiments.

**Supplementary Table S10.** List of antibodies.

**Supplementary Table S11.** List of primers.

**Supplementary Table S11.** Continued.

**SUPPLEMENTARY METHODS**

**High performance liquid chromatography of unsaturated disaccharides of GAGs.**

Disaccharide composition analysis of HS and DS was performed by a reversed phase ion-pair chromatography with sensitive and specific post-column detection. A gradient was applied at a flow rate of 1.0 ml/min on Senshu Pak Docosil (4.6 × 150 mm; Senshu Scientific, Tokyo, Japan) at 60℃. The eluent buffers were as follows: A, 10 mM tetra-*n*-butylammonium hydrogen sulfate in 12% methanol; B, 0.2 M NaCl in buffer A. The gradient program for HS analysis was as follows: 0-10 min (1-4% B), 10-11 min (4-15% B), 11-20 min (15-25% B), 20-22 min (25-53% B), and 22-29 min (53% B). The gradient program for CS analysis was as follows: 0-10 min (1% B), 10-11 min (1-10% B), 11-30 min (10% B), 30-35 min (10-60% B), and 35-40 min (60% B). Aqueous (0.5% (w/v)) 2-cyanoacetamide solution and 1 M NaOH were added to the eluent at the same flow rates (0.5 ml/min) by using a double plunger pump. The effluent was monitored fluorometrically (excitation, 346 nm; emission, 410 nm).

Where in DS, ΔDi-0S is deoxy-α-L-*threo*-hex-4-enopyranosyluronic acid (ΔUA) (1→3) *N*-acetylgalactosamine (GalNAc), ∆Di-4S is ΔUA (1→3) GalNAc4S, where S is sulfo, ∆Di-6S is ΔUA (1→3) GalNAc6S, ∆Di-UA2S is ΔUA2S (1→3) GalNAc, ∆Di-diS_E_ is ΔUA (1→3) GalNAc4S6S, ∆Di-diS_B_ is ΔUA2S (1→3) GalNAc4S, ∆Di-diS_D_ is ΔUA2S (1→3) GalNAc6S, and ∆Di-TriS is ΔUA2S (1→3) GalNAc4S6S, and in HS, ΔDi-0S_HS_ is ΔUA (1→4) *N*-acetylglucosamine (GlcNAc), ∆Di-NS is ΔUA (1→4) GlcNS, ∆Di-6S_HS_ is ΔUA (1→4) GlcNAc6S, ∆Di-NS6S is ΔUA (1→4) GlcNS6S, ∆Di-NSUA2S is ΔUA2S (1→4) GlcNS, and ∆Di-TriS_HS_ is ΔUA2S (1→4) GlcNS6S. Since ∆Di-0S (coming from DS, where ΔDi-0S is deoxy-α-L-*threo*-hex-4-enopyranosyluronic acid (ΔUA) (1→3) *N*-acetylgalactosamine (GalNAc) ) and ∆Di-HA (coming from hyaluronic acid, where ∆Di-HA is ΔUA (1→3) *N*-acetylglucosamine (GlcNAc) ) could not be separated under our HPLC conditions, a graphitized carbon column was used to separate ∆Di-0S and ∆Di-HA^1^. Expression levels of DS and HS were expressed as total amount of unsaturated disaccharides. Composition of unsaturated disaccharides of GAGs was shown in Supplementary Table S4-S7 and S9.

**High performance liquid chromatography of polyamines.**

A HPLC analysis with post-column derivatization was performed by the according to the methods of Igarashi *et al*^2^ with minor modifications. Skin samples were homogenized with 5% trichloroacetic acid and heated at 70℃ for 30 min, and then polyamines were extracted by the centrifugation at 10,000 × g. The isocratic tandem-mode HPLC system, in which two TSK gel polyaminepak columns (4.6 mm I.D × 5 cm; Tosoh Corporation) were connected in series. The isocratic elution conditions were as follows: eluent (pH 5.5), 2.0 M NaCl, 93.6 mM of trisodium citrate dihydrate, 0.01% hexanoic acid, 0.1% Brij-35 in 20% methanol; flow rate, 0.21 mL/min. To the effluent, fluorescence detection reagent including 0.4 M boric acid, 0.35 M KOH, 0.2% 2-mercaptoethanol, 0.1% Brij-35, 4.18 mM *o*-phthalaldehyde (OPA) in 0.625% methanol was delivered using double-plunger pump. The eluent was monitored fluorometrically (Ex. 340 nm, Em. 455 nm). Polyamine contents in skin and cultured cells were shown in Supplementary Table S2, S3 and S8.

**SUPPLEMENTARY REFERENCES**

1 Higashi, K. *et al.* Composition of Glycosaminoglycans in Elasmobranchs including Several Deep-Sea Sharks: Identification of Chondroitin/Dermatan Sulfate from the Dried Fins of Isurus oxyrinchus and Prionace glauca. *PLoS One* **10**, e0120860, doi:10.1371/journal.pone.0120860 (2015).

2 Igarashi, K. *et al.* Formation of a compensatory polyamine by Escherichia coli polyamine-requiring mutants during growth in the absence of polyamines. *J Bacteriol* **166**, 128-134 (1986).
